# Cup orientation following posterior approach THA – the effect of different visual aids and pelvic supports

**DOI:** 10.1186/s12891-022-05820-w

**Published:** 2022-09-22

**Authors:** Moritz M. Innmann, Jeroen Verhaegen, Christian Merle, Paul E. Beaulé, Geert Meermans, George Grammatopoulos

**Affiliations:** 1 Division of Orthopaedic Surgery, The Ottawa Hospital, Critical Care Wing, 501 Smyth Road, Ottawa, Ontario K1H 8L6 Canada; 2 Department of Orthopaedics and Trauma Surgery, University of Heidelberg, Schlierbacher Landstrasse 200a, 69118 Heidelberg, Germany; 3 Department of Orthopaedics and Traumatology, University Hospital Antwerp, Wilrijkstraat 10, 2650 Edegem, Antwerp, Belgium; 4 Orthopedic Center Antwerp, Kielsevest 14, 2018 Antwerp, Belgium; 5 Department of Orthopaedics, Bravis Hospital, Boerhaavelaan 25, 4708 AE Roosendaal, The Netherlands

**Keywords:** Hip, Arthroplasty, Pelvis, Position, Posterior approach, Cup

## Abstract

**Introduction:**

This study aims to compare cup inclination achieved (1) Using two orientation guides, whilst using the same 3-point pelvic positioner and (2) Using two types of pelvic positioners, whilst measuring intra-operative cup inclination with an inclinometer.

**Materials and methods:**

This is a prospective, diagnostic cohort study of a consecutive series of 150 THAs performed through a posterior approach. Two types of 3-point pelvic positioners were used (Stulberg and modified Capello Hip Positioners) and the cup was positioned freehand using one of two orientation guides (mechanical guide or digital inclinometer). Intra-operative inclination was recorded, radiographic cup inclination and anteversion were measured from radiographs. The differences in inclination due to pelvic position (ΔPelvicPosition) and orientation definitions (ΔDefinition) were calculated. Target radiographic inclination and anteversion was 40/20° ± 10°.

**Results:**

There was no difference in radiographic cup inclination/ (*p* = 0.63) using a mechanical guide or digital inclinometer. However, differences were seen in ΔPelvicPosition between the positioners ((Stulberg: 0° ± 5 vs. Capello: 3° ± 6); *p* = 0.011). Intra-operative inclination at implantation was different between positioners and this led to equivalent cases within inclination/anteversion targets (Stulberg:84%, Capello:80%; *p* = 0.48).

**Conclusions:**

With the pelvis securely positioned with 3-point supports, optimum cup orientation can be achieved with both alignment guides and inclinometer. Non-optimal cup inclinations were seen when intra-operative inclinations were above 40° and below 32°, or the ΔPelvicPosition was excessive (> 15°; *n* = 2). We would thus recommend that the intra-operative cup inclination should be centered strictly between 30° and 35° relative to the floor. Small differences exist between different type of pelvic positioners that surgeons need to be aware off and account for.

## Introduction

Acetabular component (cup) orientation is associated with outcome following hip arthroplasty [1–3]. The resultant cup orientation is dependent upon (a) the pelvic position at time of cup impaction; and (b) the orientation of the cup provided by surgeon during impaction [4–7]. When operating in the lateral position, great variability in the use of pelvic support exists [8, 9]. The utilization of positioners with 3-point support is associated with less intra-operative movement [6]. However, 3-point support may be achieved with a number of commercially available supports [8]; whether all provide similar degrees of constraint is unknown.

The ability of surgeons to judge inclination angles in the lateral decubitus position varies significantly [10]. To aid surgeons, the use of mechanical aids has been advocated. However, these aids vary by implant and may lead to high inclination values [11]. As such many have advocated the use of an inclinometer to better quantify intra-operative inclination and improve radiographic cup inclinations [7, 12, 13].

The aims of this study were to compare radiographic cup inclination achieved (1) Using two types of orientation guides (inclinometer or alignment guide), whilst using the same 3-point pelvic positioner; and (2) Using two types of pelvic positioners, whilst measuring intra-operative cup inclination with an inclinometer. In doing so, factors leading to cup mal-orientation were identified.

## Materials and methods

This is a prospective, institutional review board (IRB)-approved, single-surgeon, consecutive, case-series from a tertiary center. The study was conducted according to the Helsinki Declaration of 2008 and all patients gave informed consent.

One hundred and fifty consecutive primary total hip arthroplasties (THAs) performed through the posterior approach that had an uncemented acetabular component fixation, were studied between 2018 and 2020. Most patients were female (*n* = 88, 59%) and mean age at arthroplasty was 61 (±15) years old. The mean BMI was 30.9 ± 6.2 and the average adipose tissue depth (thickness of adipose tissue measured between skin and fascia lata at the level of the greater trochanter measured with a sterile ruler) was 6.7 ± 2.7 cm. Uncemented THAs were implanted in 116 cases and hybrid fixation in 34. The mean cup size was 50 ± 4 mm. Patient demographics and diagnoses are included in Table 1.

**Table 1:** Demographics and diagnoses

	Number of Hips	Mechanical alignment guide	Inclinometer	***p***-value	Stulberg positioner	Capello positioner	***p***-value
Demographics							
Number of hips	150	50	50	–	50	50	–
Gender (m: f)	62: 88	19: 31	23: 27	0.42	23: 27	20: 30	0.55
Age at surgery in years (mean, SD)	61 (15)	59 (13)	59 (18)	0.76	59 (18)	65 (13)	0.07
BMI in kg/m^2^ (mean, SD)	31 (6)	28 (5)	30 (6)	0.14	30 (6)	32 (6)	0.13
Fat thickness in cm (mean, SD)	7 (3)	7 (2)	7 (3)	0.99	7 (3)	7 (2)	0.99
Diagnosis							
Primary osteoarthritis	113	41	39	–	41	46	–
Secondary osteoarthritis due to							
Developmental dysplasia	19	5	4	–	5	2	–
Avascular necrosis	6	1	2	–	2	1	–
Other (e.g fracture, Perthe’s, failed ORIF)	12	3	5	–	2	1	–
Acetabular Implants							
CSF (JRI, London UK)	64	–	–	–	–	–	–
Trident (Stryker Orthopaedics, Mahwah, NJ, USA)	11	–	–	–	–	–	–
G7 (Zimmer Biomet, Warsaw, IN, USA)	75	–	–	–	–	–	–

### Pelvic position & support used

**Fig. 1:**
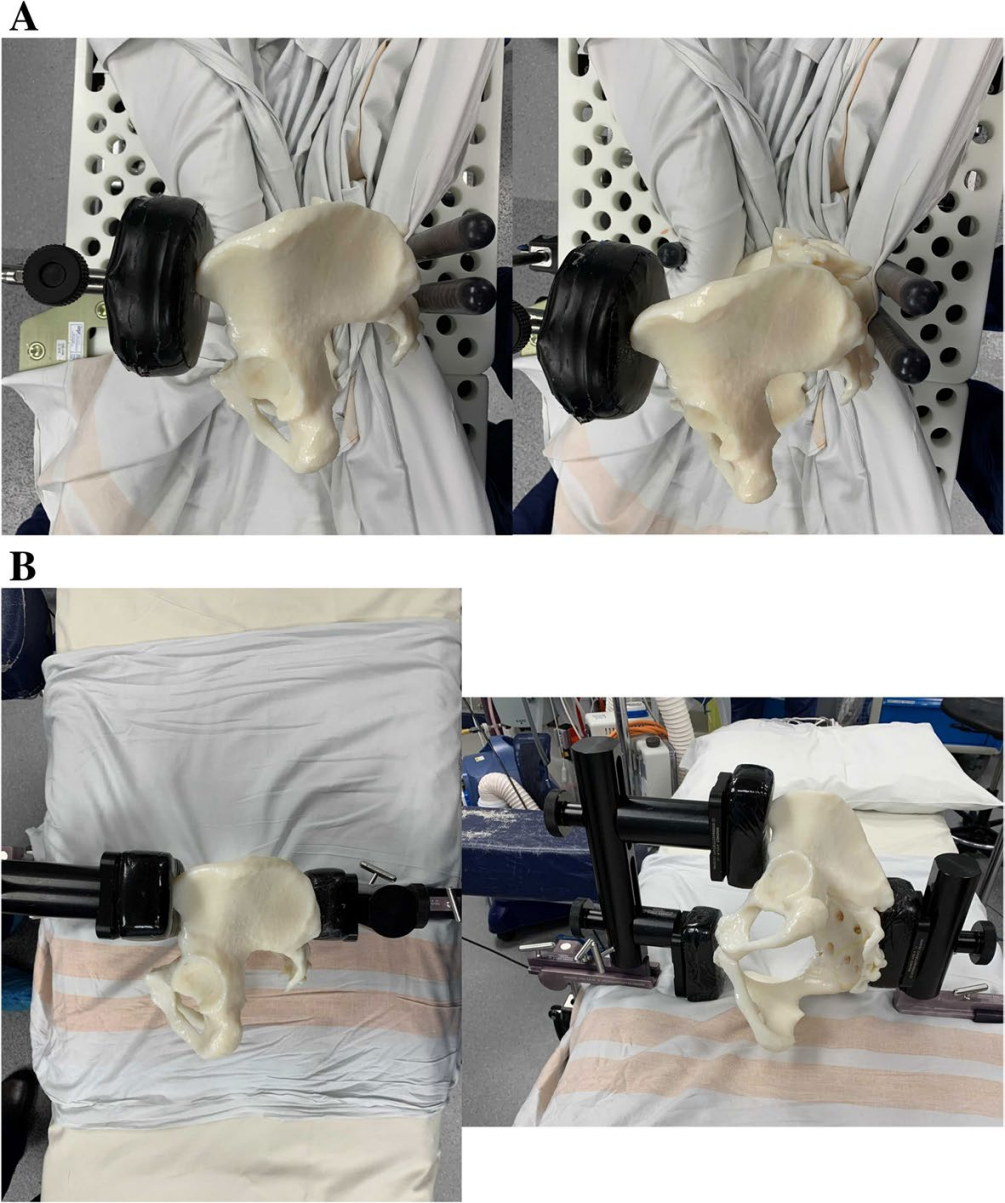
**A** Photograph illustrating pelvic positioning with the Capello Hip Positioner (pegboard with a modification, which is a single extra support over the operated Anterior Superior Iliac Spine). **B** Photograph illustrating pelvic positioning with the Stulberg Hip Positioner (no pegboard and three-point positioning system)

Two different types of pelvic positioners were used as per availability which depended on the hospital site the surgery was performed at. There were no demographic differences (including diagnosis and thickness of fat) between groups. The 2 pelvic positioners used were the Stulberg Hip Positionerand the Capello Hip Positioner (both Innomed Inc., Savannah, GA, USA). As the Capello Hip Positioner may not provide enough support on the anterior superior iliac spine (ASIS) on the operated side, an additional bolster was used as illustrated (Fig. 1) in all cases. Of the 150 cases; 100 were performed with the Stulberg and 50 were performed with the Capello Hip Positioner. All cases were positioned in the lateral decubitus position with the aim of having the operated hemi-pelvis as close to a neutral position, i.e. ‘perpendicular’, relative to the table in all planes at the time of cup impaction as possible. Given that the pelvis moves during surgery with the operated hemi-pelvis typically rotating internally during the posterior approach, the pelvis was aimed to be set with a slight externally rotated position of the operated hemi-pelvis (e.g. achieved with placing 2 blocks on the operated ASIS and 1 on the non-operated ASIS with the Stulberg Hip Positioner and visually with the Capello Positioner) [6, 7, 14, 15].

### Acetabular preparation & impaction

**Fig. 2:**
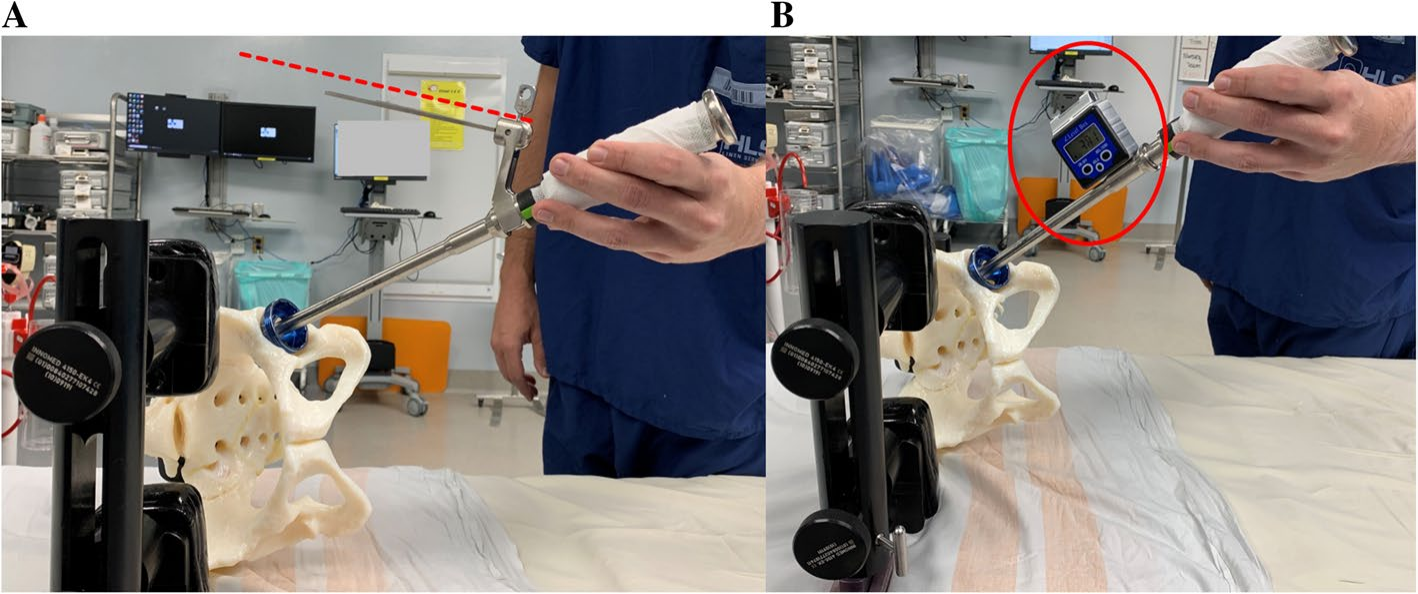
**A **&** B** Photograph illustrating the two visual cues used in this study (alignment guide – **A** and inclinometer – **B**) aligned with the impaction handle of the acetabular component

It was ensured that the patient was securely positioned and that the operating table was level. A posterior approach was performed in all procedures. The acetabular component anteversion was placed taking into consideration, the orientation of the transverse acetabular ligament (TAL), the version of the femoral component and the combined anteversion value as femoral preparation proceeded that of the acetabulum. The acetabular component inclination was positioned freehand using one of 2 visual aids; a mechanical guide as per manufacturer set at inclination/anteversion: 45/20° in 50 consecutive THAs, or an inclinometer device (Digital Protractor Angle Gauge Finder, TEKCOPLUS, ASIN: B01HBIANG4) in the remaining 100 consecutive THAs [12, 16] (Fig. 2A&B). For all cases, it was aimed that the intra-operative inclination was less than 45° (i.e. guide not parallel to floor but pointing towards ceiling or measured inclination with inclinometer ≤45°) as this has been shown to lead to higher post-operative radiographic inclinations [17]; furthermore, a strict inclinometer reading target range was not utilized as in previous studies [7, 12, 13]. The inclination angle was not adjusted as per adipose tissue thickness or BMI [12]; however, whenever a varus femoral stem was used (*n* = 6), low inclination values were avoided as best as possible to reduce incidence of impingement in flexion and abduction as per technical guide [16]. Target cup orientation zone was defined as a radiographic inclination/anteversion of 40/20° ±10° [3, 18].

### Parameters measured

The measured intra-operative inclination (IOI) angle was recorded from the inclinometer reading prior to disengaging the cup introducing handle, having ensured good cup fixation [12]. The IOI is the acetabular inclination of the cup/impactor relative to the operating table/floor. Radiographic cup inclination (RI) and anteversion (RA) was measured from post-operative, supine, anteroposterior (AP) pelvis radiographs obtained at the 6-week follow-up appointment, using the EBRA software (EBRA-cup; University of Innsbruck, Innsbruck, Austria) by an independent observer not involved with the care of these patients. The cup orientations of 15 cases (10%) were measured twice by the same reader and by a second reader to test intra- and inter- observer reliability. Average-measure correlation coefficients with a two-way random effects model for absolute agreement were calculated, showing excellent intra- and inter-observer reliabilities for radiographic measurements (range: 0.901 (95% CI; 0.705–0.969) to 0.932 (95% CI; 0.796–0.979).

### Parameters calculated

Having established the radiographic cup orientation from the post-operative AP pelvic radiograph using the pelvic co-ordinate system, the true operative cup inclination (TOI) was determined using Murray’s nomograms [4]. The TOI is the operative inclination relative to the native pelvis that the surgeon impacted the cup at (Sin (operative inclination) = Sin (radiographic inclination) * Cos (radiographic anteversion), therefore TOI = ArcSin (Sin (RI) * Cos (RA)). The difference between the true operative inclination and the intra-operative inclination reflects the deviation of the pelvic position at the time of impaction from the desired position, as defined by the radiographic assessment, (ΔPelvicPosition = TOI – IOI). The inherent difference between the radiographic and true operative inclination has been described by Murray’s definitions and is dependent on the cosine function of operative anteversion (ΔDefinition = RI – TOI).

### Analyses performed

Measurements of interest were the radiographic cup orientations achieved, the percentage of cases within target orientation, ΔPelvicPosition and ΔDefinition. We tested for potential differences in these measurements between cases with different types of pelvic positioner and different types of visual aids. To account for pelvic support and visual aids, the effect of visual aid used was only tested in cases that the Stulberg positioner was used for (*n* = 100). Similarly, the effect of positioner was only tested for cases that IOI was measured with the inclinometer (*n* = 100).

### Statistics

We wanted to be able to detect a minimum difference in pelvic movement for abduction or adduction in the coronal plane of 5° between the two different positioner systems. We therefore performed a sample size calculation a priori, based upon previously published data on intraoperative pelvic movement with different supports, resulting in a minimum cohort size of 41 patients per group (α = 0.05, 1-β = 0.95, mean difference of 5° between groups (SD6°) [6, 19]. Furthermore, a ΔPelvicPosition greater than 10° was considered significant as the size of most safe zone described are of a ± 10° margin [1, 18, 20]. Variability was defined as 2 × standard deviations (SD). Non-parametric tests (Mann–Whitney U test, Spearman’s rho) were used. The chi-squared test was used for cross-tabulated data. Statistical significance was defined as a *p* ≤ 0.05. Analyses were performed with SPSS Statistics version 26 (IBM Corp., Armonk, New York).

## Results

**Fig. 3:**
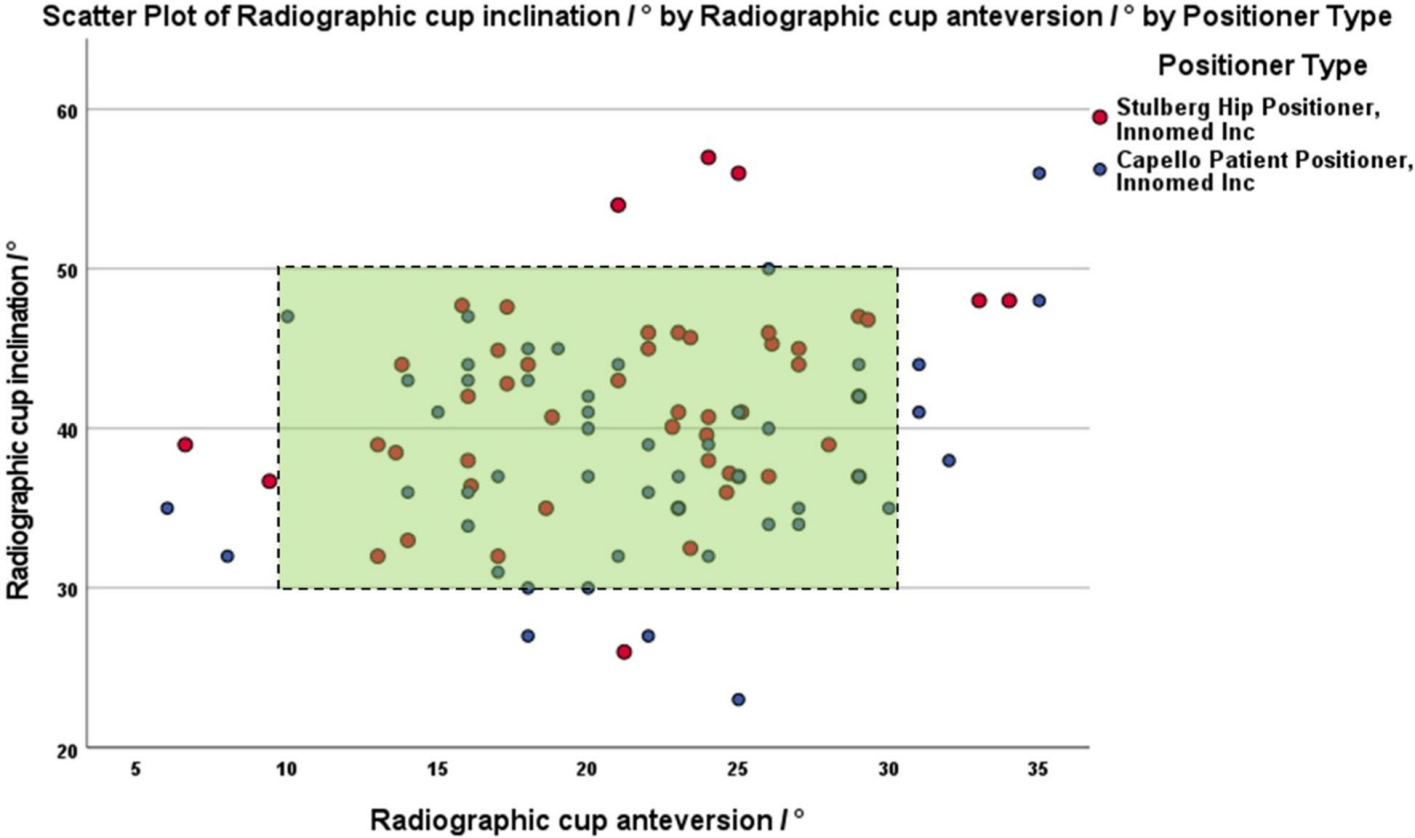
Scatterplot illustrating the cup orientations achieved (as measured with the EBRA software for inclination and anteversion (green area indicating the target cup orientation zone of inclination/anteversion of 40/20° ±10°). The graph is color coded for the two pelvic positioners used in the study

The cohort’s mean radiographic inclination was 41° ± 7 (range: 23–59). Optimum inclination was achieved in 138 THAs (92%) (Table 2 and Fig. 3). For cases that the inclinometer was used; the mean IOI was 35° ± 4 (range: 24–46) and the mean TOI was 7° ± 5 (range: 21–50). The mean ΔPelvicPosition was 2° ± 5 (range: − 13 to 16). The mean ΔDefinition was 4° ± 2 (range: 0–13) (Fig. 3).

**Table 2:** Intraoperative and radiographic measurements for all patients

Parameter	Value
Radiographic inclination	41° ± 7
Radiographic anteversion	22° ±6
Optimum inclination (30°-50°) (n,%)	138 (92%)
Optimum anteversion (10°-30°) (n,%)	138 (92%)
Optimum inclination and anteversion (n,%)	126 (84%)
Intra-operative inclination (IOI)	35° ± 4
True operative cup inclination (TOI)	37° ± 5
ΔPelvicPosition (= TOI – IOI)	2° ± 5
ΔDefinition (= RI – TOI)	4° ± 2

Accounting for the pelvic positioner used (Stulberg), there appeared to be no clinically significant difference in the radiographic cup inclination (42° ± 6 vs. 42° ± 6; *p* = 0.63) or anteversion (23° ± 5 vs. 22° ± 5; *p* = 0.32) obtained for the two visual aids used. The TOI was similar whether the mechanical alignment guide (38° ± 5) or inclinometer (37° ± 5) was used (*p* = 0.9). There was no difference for the distribution of cups with optimum orientation for the two different visual aids used (44/50 vs. 42/50; *p* = 0.56) (Table 3).

**Table 3:** Intraoperative and radiographic measurements for the two different cup orientation aids with the Stulberg positioner (an inclinometer was used in all patients with the Capello positioner) and for the two different positioners accounting for visual aid (inclinometer)

Parameter	Inclinometer (*n* = 50)	Mechanical alignment guide (*n* = 50)	*p*-value	Stulberg positioner (*n* = 50)	Capello positioner (*n* = 50)	*p*-value
Radiographic inclination	42° ± 6	42° ± 6	0.63	42° ± 6	39° ± 6	0.02
Radiographic anteversion	22° ± 5	23° ± 5	0.32	22° ± 6	22° ± 7	0.98
Optimum inclination (30°-50°) (n)	46 (92%)	46 (92%)	1.0	46 (92%)	46 (92%)	1.00
Optimum inclination and anteversion (n)	42 (84%)	44 (88%)	0.57	42 (84%)	40 (80%)	0.60
Intra-operative inclination (IOI)	37° ± 3	–	–	37° ± 3	32° ± 3	< 0.001
True operative cup inclination (TOI)	37° ± 5	38° ± 5	0.9	37° ± 5	36° ± 6	0.015
ΔPelvicPosition (= TOI – IOI)	0° ± 5	–	–	0° ± 5	3° ± 6	0.011
ΔDefinition (= RI – TOI)	4° ± 2	–	–	4° ± 2	4° ± 2	0.355

Account for the visual aid used, differences in the pelvic positioners were identified (Table 3). Although, ΔDefinition was similar between positioners the Stulberg Group (4° ± 2) compared to the Capello (4° ± 2) (*p* = 0.355), there was a significant difference in ΔPelvicPosition (Stulberg: 0° ± 5 vs. Capello: 3° ± 6; *p* = 0.011). There were less cases with significant ΔPelvicPosition in the Stulberg (3/50) compared to the Capello group (10/50) (*p* = 0.037).

**Fig. 4:**
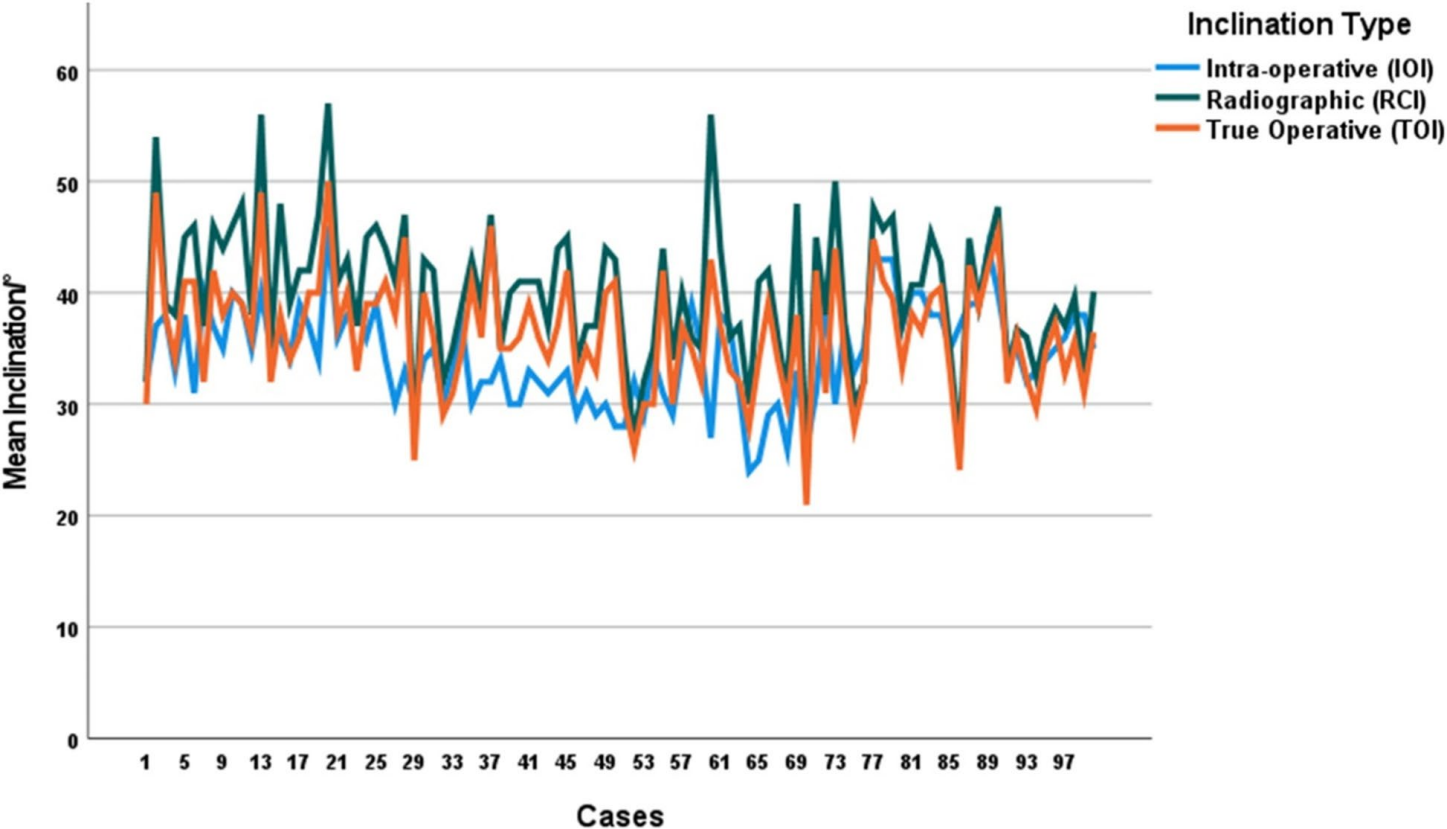
Multiple line, color-coded, diagram illustrating the intra-operative, radiographic and true operative cup inclination values for each hip arthroplasty performed with the use of an inclinometer. Data provided can be used to identify underlying cause for inclination being out of target for each of the 100 cases in the group. Identifying the difference in value for each of the cases in the study. By assessing the angular differences between TOI (orange line) and IOI (blue line) (i.e. measure of ΔPelvicPosition) and the differences between RI (green line) and TOI (orange line) (i.e. measure of ΔDefinition), one can determine what was the underlying cause for each of the outliers seen in this study

**Fig. 5:**
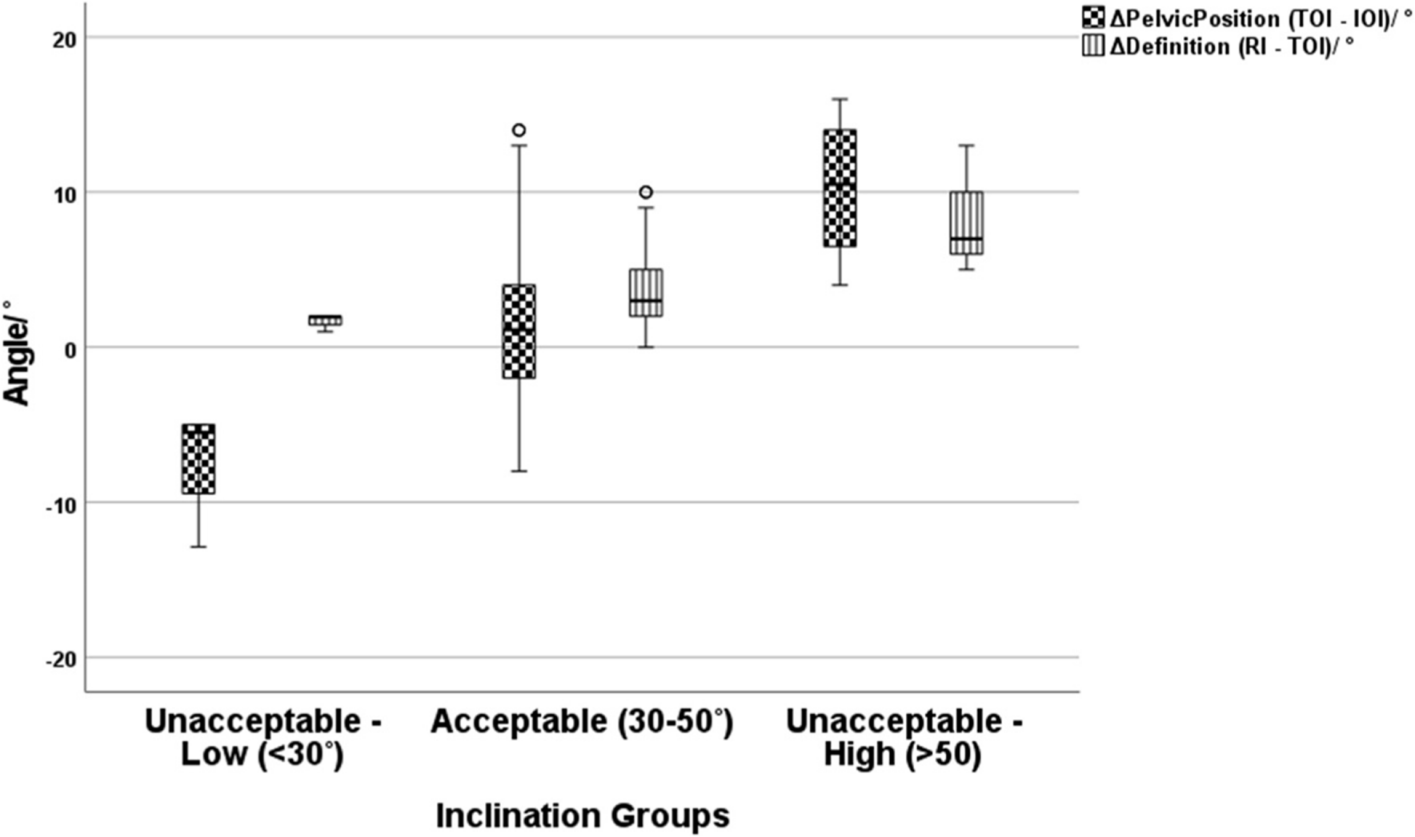
Box and whisker plot illustrating the difference ΔPelvicPosition and ΔDefinition for the three cup inclination groups (Unacceptable – low; Acceptable and Unacceptable – High). The range of ΔPelvicPosition is much greater (−13° to + 16°) than the range of ΔDefinition as seen in graph (0 to + 13°) amongst all groups

There were 8 out of the 100 inclinometer cases with sub-optimal inclinations. Four THAs had high inclinations (54°, 56°, 56°, 57°), with intra-operative inclinations that were 37°, 27°, 40° and 46°, respectively. Four THAs had low inclinations (23°, 26, 27°, 27°); with intra-operative inclinations that were 26°, 30°, 32° and 32°, respectively (Fig. 4 and Table 4). Cup mal-orientation were associated with both ΔPelvicPosition (− 7° ± 4 vs. 2° ± 5 vs. 10° ± 5; *p* < 0.001) and ΔDefinition (2° ± 5 vs. 4° ± 2 vs. 8° ± 4; *p* < 0.001) but the effect of ΔPelvicPosition was greater (Fig. 5).

**Table 4:** Cross-Tabs for cups with optimum intraoperative cup inclination angles (inclinometer reading between 33° and 39°) and optimum radiographic measurements for cup inclination (between 30° and 50° (*p*-value 0.086))

	Intraoperative Cup Inclination between 33° to 39°	Intraoperative Cup Inclination not between 33° to 39°
Optimum Postoperative Radiographic Cup Inclination (30° to 50°)	52	40
Not Optimum Postoperative Radiographic Cup Inclination (less than 30° or more than 50°)	2	6

## Discussion

In this prospective study, the mean radiographic inclination (41°) achieved were as per target of 40° and the variability was in line with previous reports (12°). By standardizing factors that have been shown to influence resultant cup orientation (surgeon, approach, and 3-point supports) we illustrated that differences exist between different pelvic supports that surgeons should be aware off. Furthermore, we tested whether the inclinometer use was associated with improved chances of obtaining target cup orientation when the pelvic is well supported. Contrary to previous reports, highlighting the beneficial effect of the inclinometer, in this study the chances of obtaining optimum orientation was similar regardless of visual aid used; however, this is likely to be the case because the inclinometer did not strictly dictate IOI as in previous studies [7, 12, 13]. Non-optimal cup inclinations were seen in cases that the intra-operative inclinations were above 40° and below 32°, or the ΔPelvicPosition was excessive (> 15°; *n* = 2). We would thus recommend that the intra-operative cup inclination should be centered about 35°, when using 3-point pelvic supports.

Operating in the lateral decubitus position is associated with greater variability in pelvic position at impaction and an associated increased variability in the cup orientations achieved [19]. In the lateral decubitus position, it is primarily the pelvic position at impaction that is the cause of the great variability seen [7, 12, 13]. Type of pelvic support is associated with degree of intra-operative movement and 3-point positioners are associated with the least intra-operative movement during a THA [6]. However, not all 3-point of pelvic positioners provide similar support. In this study, the Stulberg positioner was associated with smaller ΔPelvicPosition (0° Vs. 3°) compared to the Capello one. This led to significantly closer orientations between the intra-operative (37° vs. 32°) and the resultant radiographic (41° vs. 39°). The resultant orientations were not as different as the ΔPelvicPosition and this was due to the intra-operative orientation differences between positioners; illustrating the surgeon likely accommodated for this effect probably due to anatomical clues used in his practice (e.g. TAL). This led to equivalent chances of achieving the radiographic cup orientation target.

The orientation of the cup at impaction and the ‘offset’ due to angular projections (Murray’s definitions) are also important in determining orientation. Two commonly described methods to assess cup inclination angle at impaction, relative to the horizontal, are mechanical alignment guides and the use of an inclinometer. Contrary to previous reports that only reported on the relationship between the intra-operative and resultant radiographic inclination [7, 12, 13], the current study also took into accounted for the resultant radiographic anteversion as this has a significant effect on the difference in cup inclinations between operative and radiographic values (ΔDefinition). There was no difference in any of the parameters measured (inclination/version/chances of reaching cup orientation targets) between the visual aid groups. This likely occurred because there was no fixed narrow target of IOI with the inclinometer, contrary to previous study [3, 4, 7, 12, 18]. Overall, an intra-operative orientation < 45° was aimed for, as radiographic inclination is greater than operative inclination, but the cups were not impacted within a narrow range as evident by the measured IOI (24–46°) post-implantation. This allowed us to study what contributes to cup mal-orientation and whether too high or too low intra-operative inclination was the primary reason for most of the mal-orientated cases. The proportion of cases with cup orientations within the pre-determined target (84%), seem to be comparable to other large series (50–88%) [3, 20–24]. Several studies have investigated freehand cup-positioning, reporting a high variability of cups within the save zone for inclianation and anteversion ranging from 26 to 71% [25]. However, whether by reducing the intraoperative range would lead to less variability akin to that reported in navigated- (87–93%) [23, 24, 26] and robotically-assisted (98–100%) [24, 27] THA requires further study.

During a posterior approach to the hip, the operated hemi-pelvis at the time of impaction is adducted and internally rotated [5, 14, 15]. Thus, in order to achieve a desired radiographic inclination, the intra-operative inclination (relative to the floor) must be reduced more than the Murray definition differences(3–4°) [3, 4, 7, 12, 18]. Non-optimal cup inclinations were seen in cases that the intra-operative inclinations were above 40° and below 32°, or the ΔPelvicPosition was excessive (> 15°; *n* = 2). All patients with low inclinations, had higher IOIs recorded; this is likely to have occurred because the operated hemi-pelvis was external rotated (or abducted) at impaction. As radiographic cup orientation is very rarely less than the intra-operative but is commonly greater (5–10°: 40%) and occasionally significantly greater (> 10°: 30%), we would recommend an intra-operative cup inclination of 35°, which is in line with other recommendations [28]. Contemporary alignment guides should therefore be redesigned [17]. Until then, inclinometers should be used as the ability to judge a 5–10° reduction in inclination angle with a rod can significantly vary [10].

This study has several limitations. Firstly, it is a single surgeon series and thus suffers from operator-related biases. However, by assessing a single surgeon we were able to account for surgical-related biases which may relate to defined optimum target, ability to judge a three-dimensional angle, cup preparation and impaction techniques and pelvic set-up [1, 6, 10, 12, 18, 20, 29]. Secondly, cases were randomly assigned to the different types of pelvic supports used according to the hospital site that the operation took place, which was not associated with any patient-related bias. This is a pragmatic situation likely to be encountered by surgeons practicing in different institutions with different resources available. Thirdly, the Capello positioner was further reinforced with additional bolster and hence the results do not apply to those that use simply the pegboard, which is therefore likely to be associated with much greater ΔPelvicPosition as it cannot easily, or securely, support both ASIS. Fourthly, we could not detect a significant difference for optimally positioned cups between the two cup orientation aids or two different positioners, which might have been due to the small study cohort size. Lastly, we did not assess for any spinal pathology (e.g. scoliosis) or degree of pelvic obliquity influencing inclination angles at impaction.

In conclusion, when the pelvis is securely positioned with 3-point supports, optimum cup orientation can be achieved with both alignment guides and inclinometer. Non-optimal cup inclinations were seen when intra-operative inclinations were above 40° and below 32°, or the ΔPelvicPosition was excessive (> 15°; *n* = 2). We would thus recommend that the intra-operative cup inclination should be centered strictly between 30° and 35° relative to the floor [7, 12]. Small differences exist between different type of pelvic positioners that surgeons need to be aware off and account for when impacting the cup.

## Data Availability

The datasets used and/or analysed during the current study is available from the corresponding author on reasonable request.

## Abbreviations

THA: Total Hip Arthroplasty
IRB: Institutional review board
BMI: Body Mass Index
SD: Standard Deviation
ORIF: Open Reduction and Internal Fixation
ASIS: Anterior superior iliac spine
TAL: Transverse acetabular ligament
IOI: Intra-operative inclination
RI: Radiographic cup inclination
RA: Radiographic cup anteversion
AP: Anteroposterior
TOI: True operative cup inclination
